# miR-206 inhibits cell proliferation, invasion, and migration by down-regulating PTP1B in hepatocellular carcinoma

**DOI:** 10.1042/BSR20181823

**Published:** 2019-05-15

**Authors:** Qian Yang, Lunli Zhang, Yuanbin Zhong, Lingling Lai, Xiaopeng Li

**Affiliations:** Department of Infectious Disease, The First Affiliated Hospital of Nanchang University, Nanchang City, Jiangxi Province 330006, P.R. China

**Keywords:** Hepatocellular Carcinoma, miR-206, PTP1B, tumor growth

## Abstract

Protein tyrosine phosphatase 1B (PTP1B) has been reported as an oncogene in hepatocellular carcinoma (HCC). However, how PTP1B is regulated in HCC remains unclear. MicroRNAs (miRNAs) are a class of small non-coding RNAs involved many biological processes including tumorigenesis. In this study, we investigated whether miRNA participated in the regulation of PTP1B in HCC. We found that miR-206, which was down-regulated during tumorigenesis, inhibited HCC cell proliferation and invasion. Overexpression of miR-206 inhibited proliferation, invasion, and migration of HCC cell lines HepG2 and Huh7. Mechanistically, we demonstrated that miR-206 directly targeted PTP1B by binding to the 3′-UTR of PTP1B mRNA as demonstrated by the luciferase reporter assay. Overexpression miR-206 inhibited PTP1B expression while miR-206 inhibition enhanced PTP1B expression in HepG2 and Huh7 cells. Functionally, the regulatory effect on cell proliferation/migration/invasion of miR-206 was reversed by PTP1B overexpression. Furthermore, tumor inoculation nude mice model was used to explore the function of miR-206 *in vivo*. Our results showed that overexpression of miR-206 drastically inhibited tumor development. In summary, our data suggest that miR-206 inhibits HCC development by targeting PTP1B.

## Introduction

Liver cancer, due to the high mortality rate, ranks the second place among all causes of cancer-related deaths [1]. Hepatocellular carcinoma (HCC) is one of the most common malignancies with a high morbidity and mortality worldwide [2]. However, the precision pathogenesis of HCC is unclear and the treatment of HCC is still challenged by the existing of distant tumor metastasis by the time of diagnosis [3].

PTP1B (protein tyrosine phosphatase 1B) is a non-receptor-type oncogenic promoter involved in growth factor signaling [4]. Multiple reports found that PTP1B is up-regulated in several types of cancers, such as breast cancer, colorectal cancer, gastric cancer, and ovarian carcinomas, leading to enhanced invasion and migration of cancer cells [5–8]. Zheng et al. reported that down-regulated expression of the PTP1B was associated with aggressive clinicopathologic features and poor prognosis in HCC [9]. However, the regulation of PTP1B in human HCC progression and invasion remains unclear.

MicroRNAs (miRNAs) are endogenous small non-coding RNAs, which regulate a broad-spectrum of genes involved in developmental and oncogene pathways at the post-transcriptional level [10]. MiRNA can participate in many cellular processes such as cell cycle, proliferation, apoptosis and metastasis [11]. Several studies have demonstrated that miRNAs play important roles in hepatocarcinogenesis and malignant transformation, regulating cell proliferation, apoptosis, migration, invasion and tumorigenesis [12,13]. Thus, miRNAs are considered as potential novel targets for various cancer therapies. miR-206 is a member of miR-1 family and specifically expressed in skeletal muscle [14]. Previous reports demonstrated that miR-206 was significantly down-regulated in HCC tissue and overexpression of miR-206 could inhibit proliferation, invasion and migration of HCC cells [15]. The detailed mechanism of miR-206 in HCC development is still not well defined.

In this study, we found that miR-206, which was down-regulated during tumorigenesis, inhibited the proliferation and invasion of HCC cells. Moreover, we demonstrated that miR-206 regulated HCC development by directly targeting PTP1B by binding to the 3′-URT of PTP1B mRNA. The regulatory effect of miR-206 was blocked by PTP1B overexpression. Furthermore, overexpression of miR-206 significantly inhibited growth of tumors in nude mice after tumor inoculation *in vivo*. Taken together, the results indicated that expression of miR-206 inhibited tumor growth and metastasis by targeting PTP1B.

## Materials and methods

### Cell lines and cell culture

The HCC cell lines HepG2, Hep3B, Huh-7, and SMMC-7721 were purchased from the cell bank of the Chinese Academy of Sciences, and normal human hepatic cell line (LO2) was maintained in our laboratory and cultured in RPMI-1640 supplemented with 10% FBS (GIBCO, New York, U.S.A.) at 37°C in a 5% CO_2_ incubator, and HEK293T cells were cultured in DMEM (Invitrogen, CA, U.S.A.) supplemented with 10% FBS (GIBCO, New York, U.S.A.).

### Real-time quantitative PCR

Total RNA was extracted using TRIzol according to the manufacturer's manual (Invitrogen, CA, USA) and then reverse transcribed using the Promega kit. SYBR Green Supermix (Takara, Dalian, China) was used for real-time quantitative PCR (RT-qPCR). miRNA expression was normalized to the level of control human U6 snRNA. The primers used for RT-qPCR are as follows: miR-206: F: 5′-GCCCGCTGGAATGTAAGGAAGT-3′, R: 5′-CCAGTGCAGGGTCCGAGGT-3′; PTP1B: F: 5′-CCAGCCAAAGGGGAGCCGTC-3′, R: 5′-CTATGTGTTGCTGTTGAACA-3′; U6: F: 5′-CCAGTGCAGGGTCCGAGGT-3′, R: 5′-TGCGGGTGCTCGCTTCGCAGC-3′; GAPDH: F: 5′-GCGCGTCGTGAAGCGTTC-3′, R: 5′-CCAGTGCAGGGTCCGAGGT-3′. Results were expressed as mean ± standard deviation (SD).

### Cell proliferation assays

HepG2/Huh7 cells transfected with the miR-206 or scramble control, PTP1B overexpression vector or control vector were seeded at a density of 2 × 103 cells per well into 96-well plates. Cell proliferation was measured at indicated time points using CCK8 kit (Dojindo Laboratories, Kumamoto, Japan) according to the manufacturer's manual. Representative results from at least three independent experiments with three replications were shown.

### Boyden chamber assay

Migration assay was performed using a Boyden chamber (Corning, NY, U.S.A.) containing an 8 μm polycarbonate membrane. Experiment was done following the manufacturer's protocol. Briefly, 1 × 106 cells were resuspended in 100 μl serum-free medium and added to the upper chamber and 600 μl RPMI-1640 medium supplemented with 10% FBS was added to the lower chamber. Cells were incubated for 24 h at 37°C in a 5% CO_2_ incubator. Migratory cells to the opposite side of membrane were fixed and stained with crystal violet. Images of five random areas were selected and migrated cells were counted to calculate cell migration ratio.

### Transfection

HepG2/Huh7 cells were transfected with miR-206 mimic or scramble control miR-NC (GenePharma, Shanghai, China) or miR-206 inhibitor (Invitrogen) using Lipofectamine 2000 (Invitrogen) following the manufacturer's protocol. Cells were cultured for 48 h before analysis or other subsequent experiments. PTP1B overexpression vector was constructed by cloning human PTP1B cDNA into pMSCV-hygro vector using the following primers: 5′-ATGGAGATGGAAAAGGAGTTCGA-3′ and 5′-TGTGTTGCTGTTGAACAGGAA-3′. The pMSCV-hygro-PTP1B clones were sequencing verified. HepG2 and Huh7 cells were transfected with pMSCV-PTP1B vector and selected with G418 for 3–4 weeks. Monoclonal cells were then selected and examined for PTP1B overexpression.

### Luciferase reporter assay

Luciferase reporter vector containing wild-type PTP1B (WT) and mutant PTP1B 3′-UTR were constructed and co-transfected with miR-206 mimics or scramble control into HEK293 cells. After 24 h, luciferase activity was measured using the Dual-Luciferase Reporter Assay System (Promega, Madison, WI, USA) according to the manufacturer′s protocol.

### Western blot

Western blot was performed as previously described [16]. Briefly, 20 μg of protein samples were separated by 10% SDS-PAGE gels, and then transferred to nitrocellulose membranes (Millipore, Madison, WI, U.S.A.). The membranes were then blocked in 5% non-fat dry milk in TBS buffer containing 0.1% Tween-20 for 2 h, and subsequently probed with the following primary antibodies: mouse anti-human PTP1B (ab201974, abcam) and mouse anti-human GAPDH (ab8245, abcam) at 4°C overnight. The membrane was further incubated with secondary HRP-conjugated goat anti-mouse IgG (ab6789, abcam) for 1 h at room temperature. The immunoreactive signals were detected using ECL kit (Cell Signaling, Danvers, MA, U.S.A.). The density of the band was quantified by Quantity One software (Bio-Rad).

### Mice and tumor model

Female BABL/c nude mice (4-week-old) were purchased from Shanghai Laboratory Animal Center (Shanghai, China) and maintained under a specific pathogen-free condition. All experiments were approved and carried out according to the guidelines of the Ethics Committee of The First Affiliated Hospital of Nanchang University. 5 × 10^6^ viable HepG2 cells stably transfected with the miR-206 mimic or control were planted into the right flanks of nude mice. Tumor sizes were measured every 7 days, and tumor volume was calculated: volume = 1/2 × length × width^2^. At 42 days after implantation, the mice were killed and tumors were dissected and analyzed.

### Statistical analysis

Data were expressed as mean ± SD. One-way ANOVA was used for analysis between multiple groups. Calculations were performed using GraphPad Prism software (V5.0, GraphPad, La Jolla, CA, USA). *P < 0.05 suggests a statistically significant difference.

## Results

### MiR-206 was down-regulated in human HCC cell lines and miR-206 overexpression significantly inhibited cell proliferation, migration, and invasion

**Figure 1:**
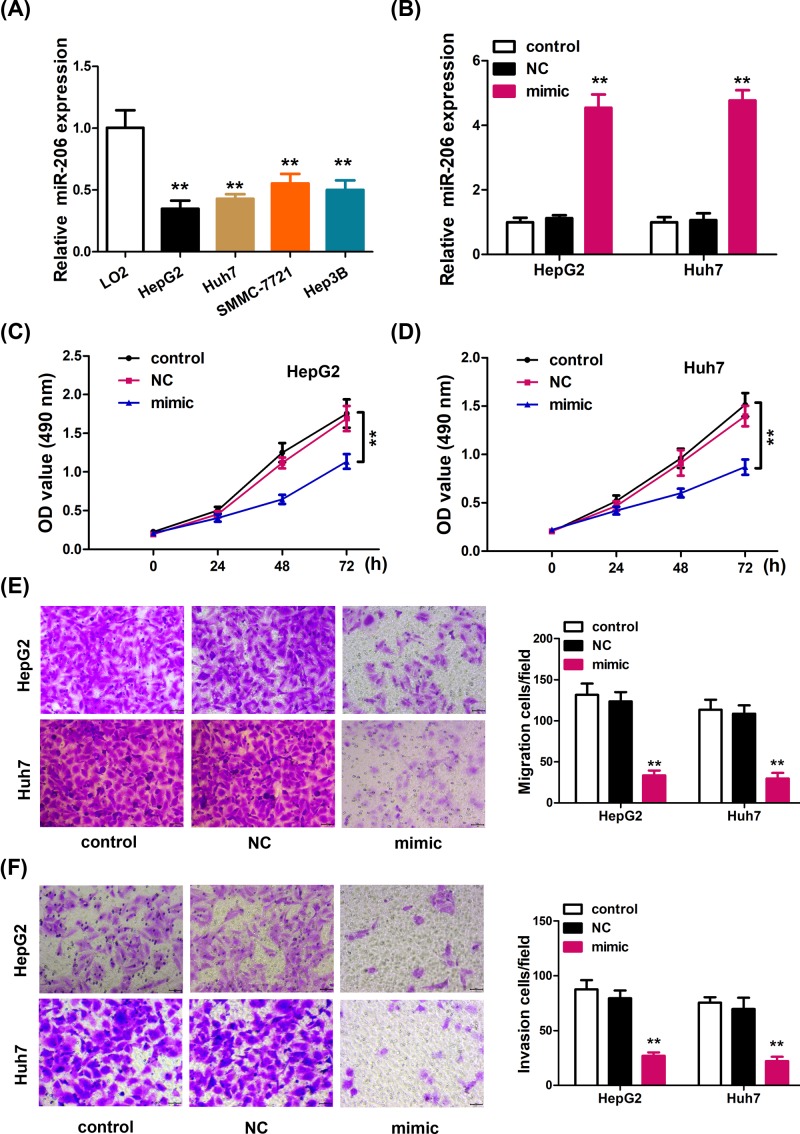
miR-206 overexpression significantly inhibited cell proliferation, migration, and invasion (**A**) Real-time PCR analysis of miR-206 expression in the normal human hepatic cell line LO2 and the HCC cell lines HepG2, Huh7, SMMC-7721 and Hep3B. The data are expressed as the mean ± SD, *n* = 6. **P < 0.01 versus the LO2 control. (**B**) Real-time PCR analysis of miR-206 expression in HepG2 and Huh7 cells transfected with miR-206 mimics, negative control (NC), or untransfected (control). The data are expressed as the mean ± SD, *n* = 6. **P < 0.01 versus control. (**C**, **D**) Ectopic expression of miR-206 significantly inhibited (**C**) HepG2 and (**D**) Huh7 cell proliferation. (**E**, **F**) Cell migration and invasion were determined in HepG2 and Huh7 cells using the Transwell (Costar, Cambridge, MA, U.S.A.) assay after transfection with the miR-206 mimic or miR-NC. Scale bar, 20 μm. Data are expressed as mean ± SD. **P < 0.01 vs. control.

To examine miR-206 expression in HCC cell line, qRT-PCR was used to quantify the miR-206 expression in the normal human hepatic cell line LO2 and HCC cell lines HepG2, Huh7, SMMC-7221, and Hep3B. The results showed that the expression of miR-206 was significantly down-regulated in all four HCC cell lines (Figure 1A). The expression levels of miR206 was much lower in HepG2 and Huh7 cells, so we used these two cell lines for the subsequent experiments. To test the function of miR-206 in HCC growth, miR-206 mimics and negative control (NC) were transfected into HepG2 and Huh7 cells. Overexpression of miR-206 was confirmed by qRT-PCR in HepG2 and Huh7 (Figure 1B). As shown in Figure 1C,D, miR-206 mimic transfection significantly inhibited the cell proliferation of HepG2 and Huh7 *in vitro*. Boyden chamber assay demonstrated that miR-206 overexpression dramatically inhibited the cell migration of HepG2 and Huh7 cells, compared with that of control group or NC group (Figure 1E). Additionally, invasion assay was performed using Matrigel. As shown in Figure 1F, ectopic expression of miR-206 significantly decreased the invasion capacity of HepG2 and Huh7 cells.

### PTP1B was a potential target of miR-206

**Figure 2:**
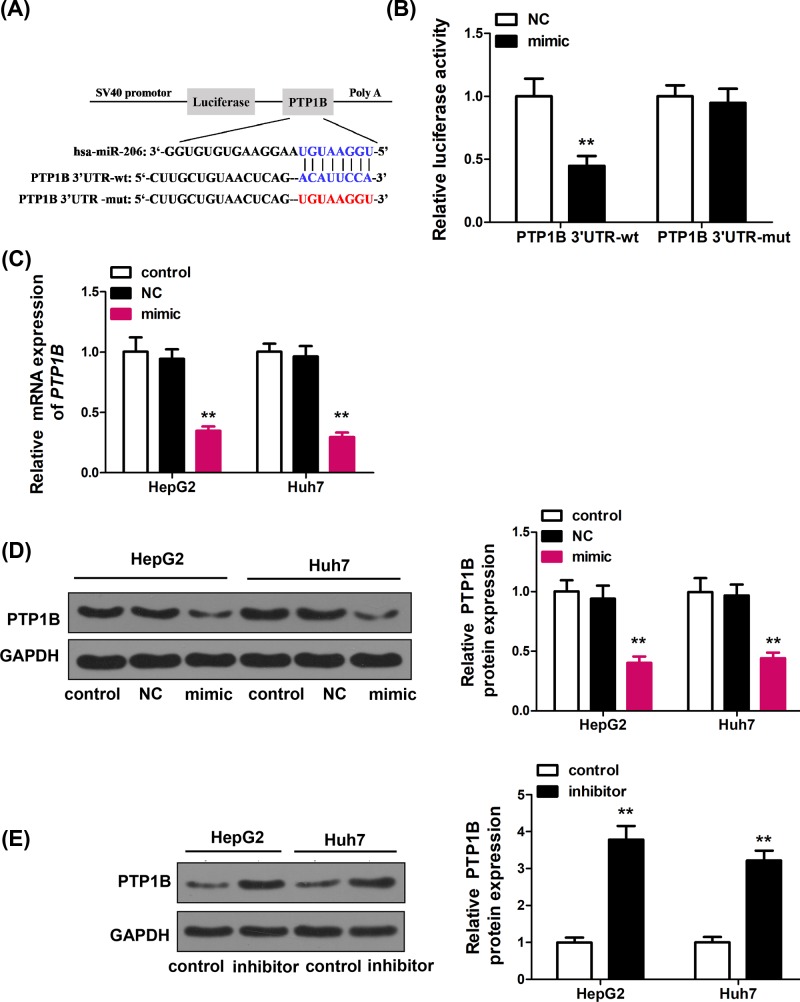
PTP1B is a potential target of miR-206 (**A**) Complementary sequences between miR-206 and the 3′-UTR of PTP1B mRNA was obtained using publicly available algorithms. The mutated version of the PTP1B 3′-UTR is also shown. (**B**) The 3′-UTR of PTP1B was fused to the luciferase coding region (PYr-PTP1B 3′-UTR) and co-transfected into HEK293T cells with miR-206 mimics to confirm that PTP1B is the target of miR-206. PYr-PTP1B 3′-UTR and miR-206 mimic constructs were co-transfected into HEK293T cells with a control vector and relative luciferase activity was determined 48 h after transfection. Data are expressed as mean ± SD. **P < 0.01 vs. NC. (**C**) RT-PCR analysis of the effect of PTP1B expression in HepG2 and Huh7 cells after transfection with miR-206 mimics (*n* = 6). GAPDH expression levels were detected as an endogenous control. Data are expressed as mean ± SD. **P < 0.01 vs. control. (**D**) Western blot analysis of the effect of PTP1B expression in HepG2 and Huh7 cells after transfection with miR-206 mimics (*n* = 6). GAPDH expression levels were detected as an endogenous control. Data are expressed as mean ± SD. **P < 0.01 vs. control. (**E**) Western blot analysis of the effect of PTP1B expression in HepG2 and Huh7 cells after treatment with miR-206 inhibitor (*n* = 6). GAPDH expression levels were detected as an endogenous control. Data are expressed as mean ± SD. **P < 0.01 vs. control.

To explore the potential molecular mechanism of miR-206 function in HCC, we searched for miR-206 targets using different prediction tools such as TargetScan, miRanda, and miRWalk algorithms. PTP1B was predicted as a potential target of miR-206. To verify whether PTP1B was a direct target of miR-206, human PTP1B 3′-UTR fragment containing the binding sites of miR-206 or the mutation sites (Figure 2A) were cloned into the pGL3 vector, and co-transfected with miR-206 mimic or NC into HEK293T cells. Relative luciferase activities were measured 48 h post transfection. The results showed that overexpression miR-206 suppressed the luciferase activity of wild-type PTP1B site, but not the vector with the mutant PTP1B site (Figure 2B), suggesting that PTP1B is directly targeted by miR-206. Additionally, qRT-PCR and western blot were performed to examine PTP1B on mRNA (Figure 2C) and protein levels (Figure 2D) in HepG2 and Huh7 cells transfected with NC or miR-206 mimic. The result showed that PTP1B mRNA and protein levels decreased in miR-206-transfected cells. Furthermore, we found that miR-206 inhibition enhanced PTP1B protein expression in HepG2 and Huh7 cells (Figure 2E). Collectively, our results suggested that PTP1B was a potential target of miR-206.

### Overexpression of PTP1B antagonizes miR-206-inhibited cell proliferation, migration, and invasion

**Figure 3:**
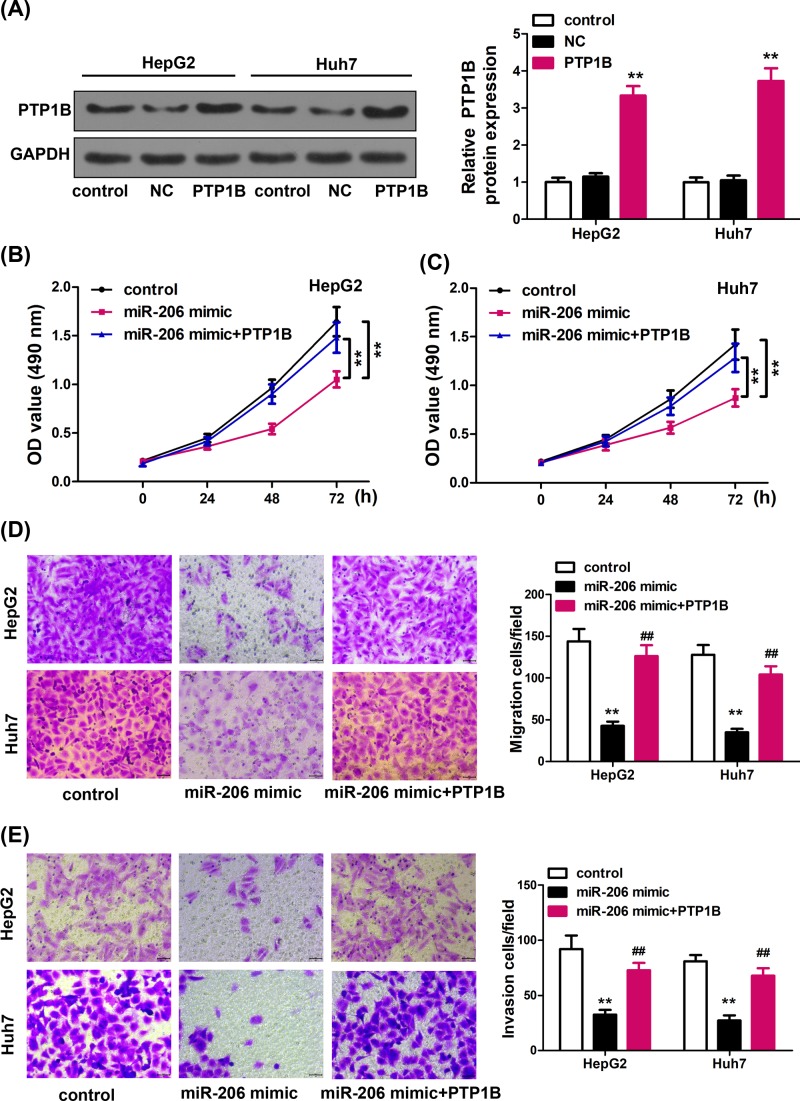
Expression of PTP1B decreased miR-206-induced cell proliferation, migration, and invasion (**A**) Western blot analysis shows the expression of PTP1B in HepG2 and Huh7 cells after transfection with the PTP1B overexpression vector or the corresponding negative control (NC) vector. (**B**, **C**) Ectopic expression of PTP1B significantly reversed miR-206-induced (**B**) HepG2 and (**C**) Huh7 cell proliferation suppression. Data are expressed as mean ± SD. **P < 0.01 vs. control. (**D**, **E**) Cell migration and invasion were determined in HepG2 and Huh7 cells using the Transwell (Costar) assay. Scale bar, 20 μm. Data are expressed as mean ± SD. **P < 0.01 vs. control.

*Ptpn1* mRNA and PTP1B protein expression was up-regulated in HCC cell lines compared with those in normal human hepatic cell line LO2 (Supplementary Figure S1). To further characterize the function of PTP1B in HCC cells and the regulation of PTP1B by miR-206, we constructed PTP1B overexpression vector and confirmed the high protein levels of PTP1B in HepG2 and Huh7 cells after PTP1B vector transfection (Figure 3A). As shown in Figure 3B,C, miR-206 mimic transfection significantly inhibited the proliferation of HepG2 and Huh7 *in vitro*, while overexpression of PTP1B together with miR-206 mimic rescued the cell proliferation.

Consistently, PTP1B overexpression in HepG2 and Huh7 cells reversed the inhibitory function of cell migration and invasion by miR-206 mimic (Figure 3D,E). Taken together, overexpression of PTP1B antagonized miR-206 inhibited cell proliferation, migration and invasion.

### miR-206 suppresses tumor growth of HCC in nude mice by inhibiting PTP1B

The previous *in vitro* results suggested that miR-206 could suppress HCC cell growth and metastasis *in vitro*. Therefore, we further investigated that whether miR-206 could affect HCC tumor growth *in vivo*. HepG2 cells were stably infected with/without the miR-206 overexpression mimic vectors and implanted subcutaneously into nude mice to allow tumor formation. Tumor sizes were measured every 7 days and mice were killed 6 weeks later.

**Figure 4:**
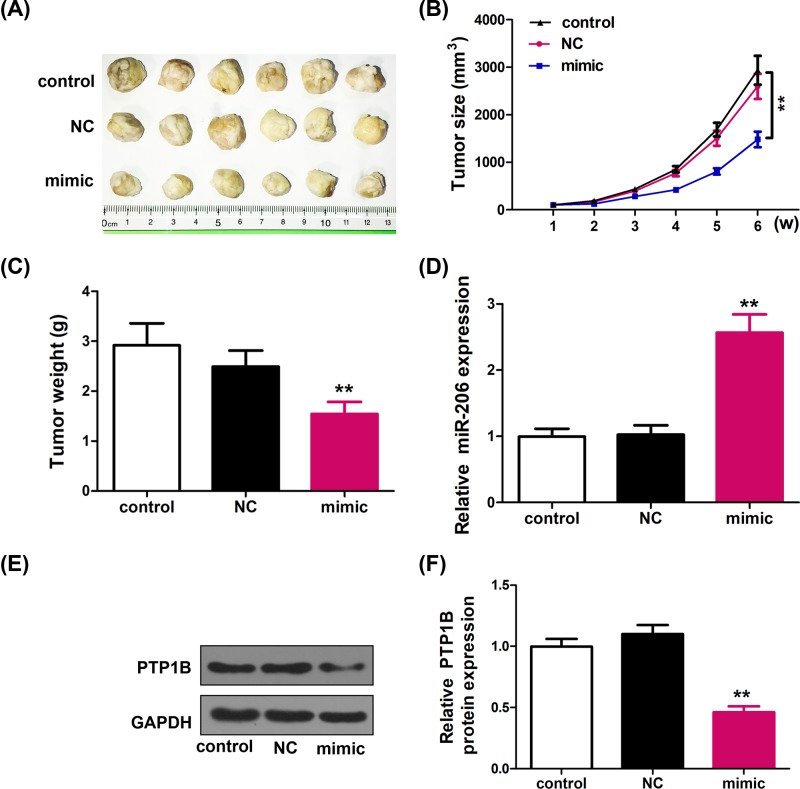
miR-206 suppressed tumor growth of HCC in a xenograft model (**A**) Photographs of tumor tissues from different groups at day 42. (**B**) Growth curves of tumor volumes in xenografts of nude mice were determined based on tumor volume measured every 7 days for 42 days (*n* = 6). **P < 0.01 vs. control group. (**C**) Tumor weights of different groups at day 42 (*n* = 6). **P < 0.01 vs. control group. (**D**) Expression of miR-206 was detected by quantitative RT-PCR (*n* = 6). Data are expressed as mean ± SD. **P < 0.01 vs. control group. (**E**, **F**) PTP1B level in tumor tissues of different groups was determined by western blot analyses. Data are expressed as mean ± SD. **P < 0.01 vs. control group.

We found that the xenograft tumor was significantly smaller in the miR-206 overexpression group compared with those in the NC group (Figure 4A). The volume (Figure 4B) and weight (Figure 4C) were also decreased in miR-206 mimic group, indicating that miR-206 overexpression suppresses HCC tumor growth *in vivo*. We also checked the miR-206 overexpression in tumor xenograft (Figure 4D). As shown in Figure 4E,F, PTP1B protein expression was down-regulated in xenograft tumors of miR-206 mimic group. These results suggested that miR-206 suppresses HCC tumor growth in nude mice by inhibiting PTP1B.

## Discussion

HCC is the fifth most common solid cancer and the third leading cause of cancer-related mortality worldwide, caused about 600000 deaths per year [1]. However, its underlying molecular mechanism remains largely unknown. Recently, several miRNAs have been reported to be involved in the development of HCC [17–20]. miR-206, which was down-regulated in HCC, was reported to suppress cell proliferation and promote apoptosis [20]. However, the mechanisms underlying miR-206 regulating HCC progress is illusive. In this study, we demonstrated that miR-206 inhibited the proliferation, migration and invasion of HCC cells *in vitro* by targeting PTP1B. *In vivo* tumor inoculation nude mice model confirmed that overexpression of miR-206 significantly inhibited growth of HCC tumors. Thus, our results suggest that miR-206 could be used not only as a novel biomarker but also as a potential gene therapeutic target of HCC.

miRNAs act as transcriptional regulators and play critical roles in HCC [21]. miR-206 is one of the most studied myomiRs, which was first reported regulating skeletal muscle development and pathology [22]. miR-206 is also involved in the pathogenesis of numerous diseases including heart failure, chronic obstructive pulmonary disease and various types of cancers [23]. According to miRNA target databases, the relationships between miRNAs and their targets might not be one-to-one [24]. Pang et al. reported that miR-206 could inhibit the growth of hepatocellular carcinoma cells via targeting CDK9 [25], while another group demonstrated that miR-206 regulates the proliferation and metastasis of HCC cells via targeting E2F5 [26]. miR-206 was also demonstrated to prevent the pathogenesis of HCC by modulating expression of met proto-oncogene and cyclin-dependent kinase 6 in mice [27]. Consistent with these findings, our results confirmed that miR-206 expression was down regulated in HCC cell lines. We used the prediction algorithms of TargetScan, miRWalk and miRanda to predict that miR-206 can target the 3′-URT of PTP1B. According to the miRNA target databases, one miRNA could target multiple genes while one gene could be regulated by multiple miRNAs [28]. It is possible that miR-206 inhibits the growth of HCC via multiple different targets. To our knowledge, miR-206 can target PTP1B regulating HCC cell proliferation, migration and invasion has not been confirmed until now.

PTP1B is a classical non-transmembrane protein tyrosine phosphatase that plays a key role in metabolic signaling and has both tumor suppressing and promoting effects in different cancers [4,29]. Liu et al. found that PTP1B promotes aggressiveness of breast cancer cells by regulating PTEN [30]. Levels of PTP1B were found to modulate the apoptotic pathways triggered by trophic factors withdrawal in hepatocytes [31]. It also has been reported that down-regulated expression of PTP1B was associated with aggressive clinicopathologic features and poor prognosis in HCC [9]. Tai et al. reported that PTP1B expression was significantly higher in HCC tumor parts than nontumor parts [32]. While PTP1b inhibitor Sorafenib was able to decrease PTP1B activity in HCC, PTP1B overexpression impaired the sensitivity of sorafenib both *in vitro* and *in vivo* [32]. The data from the present study showed that PTP1B was a direct target of miR-206 and overexpression PTP1B could antagonize miR-206-inhibited HCC cell proliferation, migration and invasion. Furthermore, PTP1B protein expression was also down-regulated in xenograft tumors after miR-206 mimic overexpression. Both *in vitro* and *in vivo* results suggested that miR-206 suppresses HCC cell proliferation, migration and invasion by inhibiting PTP1B.

In conclusion, our study demonstrated that miR-206 expression was decreased in HCC cell lines and miR-206 can inhibit cell proliferation, migration and invasion *in vitro*, as well as suppress tumor growth *in vivo* by targeting PTP1B. Furthermore, overexpression PTP1B could antagonize the inhibitory function of miR-206. These findings suggest that the miR-206 is a potential tumor suppressor in HCC, which might be a promising target for HCC treatment.

## Supporting information

**Supplementary Figure S1:** 

## Abbreviations

HCC: hepatocellular carcinoma
miRNA: microRNA
NC: negative control
PTP1B: protein tyrosine phosphatase 1B
RT-qPCR: real-time quantitative PCR
