# Supplementary figures and images for: miR-206 inhibits cell proliferation, invasion, and migration by down-regulating PTP1B in hepatocellular carcinoma
Source: Biosci Rep. 2019 May 15;39(5):BSR20181823. doi: 10.1042/BSR20181823 (PMC6522750; doi:10.1042/BSR20181823)

**A**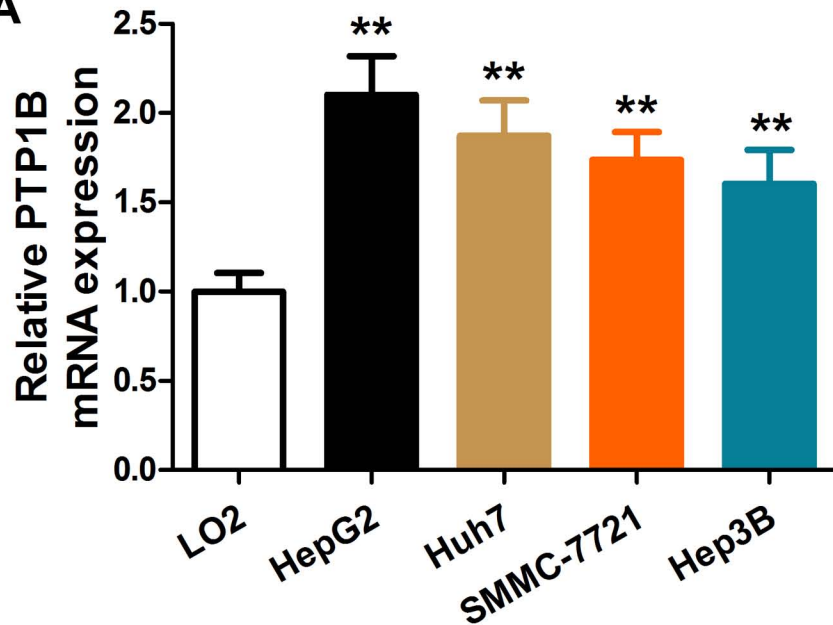**B**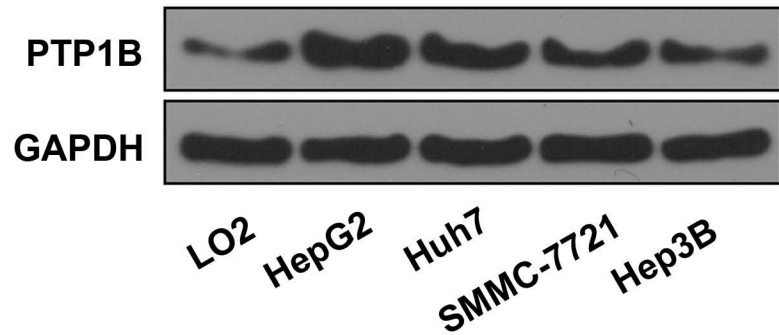

Supplement: Supplementary file 1 [file BSR-2018-1823_suppS1.pdf]
